# Pulmonary transplantation of alpha-1 antitrypsin (AAT)-transgenic macrophages provides a source of functional human AAT in vivo

**DOI:** 10.1038/s41434-021-00269-3

**Published:** 2021-07-19

**Authors:** Ewa Janosz, Miriam Hetzel, Hanna Spielmann, Srinu Tumpara, Charlotte Rossdam, Marc Schwabbauer, Doreen Kloos, Constantin von Kaisenberg, Axel Schambach, Falk F. R. Buettner, Sabina Janciauskiene, Nico Lachmann, Thomas Moritz

**Affiliations:** 1 Institute of Experimental Hematology, Hannover Medical School, Hannover, Germany; 2 REBIRTH – Research Center for Translational Regenerative Medicine, Hannover, Germany; 3 Department of Internal Medicine, Biomedical Research in Endstage and Obstructive Lung Disease Hannover (BREATH), Member of the German Center for Lung Research (DZL), Hannover Medical School, Hannover, Germany; 4 Institute of Clinical Biochemistry, Hannover Medical School, Hannover, Germany; 5 Department of Obstetrics and Gynecology, Hannover Medical School, Hannover, Germany

**Keywords:** Respiratory tract diseases, Cell delivery, Haematopoietic stem cells, Genetic transduction, Gene therapy

## Abstract

Inherited deficiency of the antiprotease alpha-1 antitrypsin (AAT) is associated with liver failure and early-onset emphysema. In mice, in vivo lentiviral transduction of alveolar macrophages (AMs) has been described to yield protective pulmonary AAT levels and ameliorate emphysema development. We here investigated the pulmonary transplantation of macrophages (PMT) transgenic for AAT as a potential therapy for AAT deficiency-associated lung pathology. Employing third-generation SIN-lentiviral vectors expressing the human AAT cDNA from the CAG or Cbx-EF1α promoter, we obtained high-level AAT secretion in a murine AM cell line as well as murine bone marrow-derived macrophages differentiated in vitro (AAT MΦ). Secreted AAT demonstrated a physiologic glycosylation pattern as well as elastase-inhibitory and anti-apoptotic properties. AAT MΦ preserved normal morphology, surface phenotype, and functionality. Furthermore, in vitro generated murine AAT MΦ successfully engrafted in AM-deficient Csf2rb^–/–^ mice and converted into a CD11c^+^/Siglec-F^+^ AM phenotype as detected in bronchoalveolar lavage fluid and homogenized lung tissue 2 months after PMT. Moreover, human AAT was detected in the lung epithelial lining fluid of transplanted animals. Efficient AAT expression and secretion were also demonstrated for human AAT MΦ, confirming the applicability of our vectors in human cells.

## Introduction

Alpha-1 antitrypsin (AAT) is an acute phase glycoprotein produced and secreted into the serum predominantly by hepatocytes and to a lesser extent by other cells, including intestinal epithelium, neutrophils, and macrophages (MΦ) [1–3]. The primary role of AAT is the inhibition of neutrophil elastase (NE) and other proteases released by activated neutrophils and other cells in the context of an inflammatory response. Moreover, AAT has been shown to have immunomodulatory and anti-apoptotic properties demonstrating its multifaceted physiologic roles [4–7]. Although NE is secreted as an antimicrobial agent in response to inflammatory stimuli, when not inhibited, it can also damage the host tissues, especially the lung, as occurs in AAT deficiency (AATD) [8, 9].

AATD may be associated with childhood and adult liver failure as well as early-onset pulmonary emphysema in the third to fourth decade of life and results from mutations in the *SERPINA1* gene encoding AAT. While many disease-causing *SERPINA1* alleles exist, the most common pathological variant, the Z (Glu342Lys) allele, leads to the production of malfunctional AAT prone to polymerization and retention of polymers within hepatocytes followed by severe hepatic disease and hepatic failure in some cases [10]. Other mutant alleles code for less-functional AAT or complete absence of the protein (null alleles). As a result, the low or absent levels of AAT in the circulation fail to protect the fragile alveolar walls from the excessive activity of NE and other proteases during inflammatory reactions, leading to the destruction of the elastin fibers and subsequently development of emphysema [11]. Currently, the only therapy available for AATD-related lung disease is substitution therapy employing weekly intravenous infusion of AAT purified from human plasma [12, 13]. However, this augmentation therapy is associated with high costs, limited availability, and the risk of transmitting pathogens. In this context, also a variety of genetic approaches aiming to provide constant production of transgenic AAT have been pursued. In particular adeno-associated viral vector (AAV)-mediated transgene delivery to the skeletal muscles has been evaluated in clinical trials, but only low levels of AAT were detectable in the serum of treated patients [14–16]. On the other hand, only ~10% of the AAT present in the serum diffuses to the epithelial lining fluid (ELF) of the lung to protect the lung tissue [8, 17]. Thus, other strategies aim for direct delivery of the transgenic AAT to the lung or pleural cavity. In line with that, intrapleural delivery of the AAT-coding AAVrh.10 vector demonstrated sustained expression of human AAT in mice and non-human primates [18] and led to the development of clinical trials [19] (NCT02168686). Other studies investigated intrapulmonary delivery of AAT-encoding lentiviral vectors (LVs) and demonstrated amelioration of elastase-induced emphysema in mice after transduction of alveolar macrophages (AMs) [20], or lung epithelial cells [21] depending on the pseudotype of the vector.

AMs, as well as other tissue-resident MΦ (TRM), have recently been described to represent a long-lived cell population originating from the early (primitive) waves of hematopoiesis during embryonic development of the organism, which under steady-state conditions will persist well into adulthood [22–24]. Physiologically, different turnover rates into definite hematopoiesis-derived cells exist for each TRM population. In case of injury or infection additional bone marrow-derived monocytes (BMDMs) can infiltrate the tissues and replenish the TRM pool, although to different extent depending on the specific organ. Also, a substitution of the AM pool by BMDMs in response to infection or radiation-mediated injury has been shown. Along the same line, intrapulmonary delivery of MΦ of different origin has demonstrated that these MΦ are able to engraft in the lung environment and develop into functional AMs, highlighting MΦ as a valuable source for cell-based therapies in the context of lung diseases [25–29].

Given this background, we here sought to evaluate primary MΦ as a source for the delivery of functional human AAT. Therefore, we employed third-generation self-inactivating (SIN) LVs to express human AAT in murine as well as human MΦs derived in vitro from bone marrow progenitor cells demonstrating functional AAT secretion from these vectors. Importantly, upon pulmonary MΦ transplantation (PMT) of murine AAT-overexpressing MΦ (AAT MΦ), the transplanted cells could be recovered from the lungs 2 months after transplantation, and human AAT was detected in the bronchoalveolar lavage fluid (BALF) of the mice. Furthermore, we show expression and secretion of human AAT from human cord blood-derived MΦ upon transduction with our lentiviral constructs, thus, confirming the applicability of our vectors in human cells.

## Results

### Efficient transgenic expression and secretion of human AAT in primary murine MΦ

**Fig. 1:**
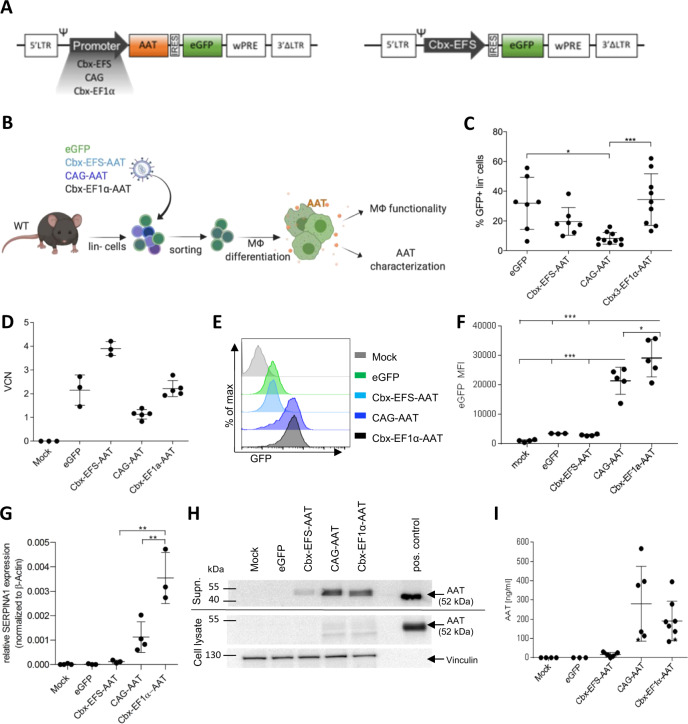
Expression of human AAT in murine MΦ. **A** Design of the third-generation, self-inactivating lentiviral vector expressing human M-type α1 antitrypsin (AAT) cDNA coupled to an enhanced green fluorescence protein (eGFP) reporter by an internal ribosomal entry site (IRES) from the Cbx-EFS, CAG, or Cbx-EF1α promoter, respectively (left). Schematic design of the control vector expressing the eGFP after an IRES sequence under the Cbx-EFS promoter (right). 5’ LTR 5’ long terminal repeat, ψ packaging signal, wPRE Woodchuck hepatitis virus posttranscriptional regulatory element, 3’ ΔLTR 3’ long terminal repeat with deletion leading to self-inactivation; EFS, EF1α: elongation factor 1α short (EFS) or long version (EF1α); Cbx: element derived from the 5’ part of the HNRPA2B1/CBX3 ubiquitous chromatin opening element, CAG: synthetic promoter composed of the cytomegalovirus early enhancer, the chicken beta-actin promoter and the splice acceptor of the rabbit beta-globin gene. **B** Scheme of the experimental procedure. Lineage negative (lin^-^) cells were isolated from bone marrow of wildtype (WT) C57BL/6J mice, transduced with AAT or eGFP control vectors, sorted for eGFP expression, differentiated into macrophages (MΦ), and subsequently used for evaluation of MΦ functionality and AAT expression as well as AAT functionality. Created with BioRender.com. **C** Percentage of eGFP^+^ cells before sorting representing the efficiency of lin^-^ cell transduction of the lentiviral vectors. **D** Determination of the vector copy number (VCN) per genome after lentiviral transduction and eGFP^+^ sorting in murine MΦ. **E** Representative histograms of the eGFP expression after lentiviral transduction and eGFP^+^ sorting in murine MΦ. **F** Median fluorescence intensity (MFI) of eGFP after lentiviral transduction and eGFP^+^ sorting in murine MΦ. eGFP *n* = 3; mock *n* = 4; Cbx-EFS-AAT, CAG-AAT, Cbx-EF1α-AAT *n* = 5. **G** Expression of human *SERPINA1* mRNA after lentiviral transduction and eGFP^+^ sorting in murine MΦ measured by RT-qPCR. **H** Representative human AAT western blot analysis of MΦ supernatant and MΦ lysates. Human serum was used as a positive control. Vinculin band in cellular lysates serves as a loading control. **I** ELISA quantification of human AAT secretion by murine MΦ. Asterisks mark the samples that were used for the representative western blot in (**H**). Each point in graphs and all *n*-numbers given represent independent biological experiments, which is an individual isolation of lin^-^ cells and individual transduction. Lines and error bars represent mean ± SD. Statistical analysis was performed using one-way ANOVA with Tukey’s post-hoc test. *p ≤ 0.05, **p ≤ 0.01, ***p ≤ 0.001.

For stable transgene overexpression in MΦ, we employed third-generation SIN LVs expressing the human healthy “M” type AAT cDNA coupled to an eGFP reporter by an internal ribosomal entry site (IRES). Three different promoter constructs were evaluated: elongation factor 1α short (EFS) or long version (EF1α) both coupled with the CBX3 ubiquitous chromatin opening element shown to improve expression levels and reduce transgene silencing [30] (referred to as Cbx-EFS-AAT and Cbx-EF1α-AAT, respectively) and the synthetic CAG promoter composed of the cytomegalovirus early enhancer, the chicken beta-actin promoter and the splice acceptor of the rabbit beta-globin gene (CAG-AAT) [31]. As a control, a LV construct expressing eGFP after the IRES sequence from the Cbx-EFS promoter was used (eGFP) (Fig. 1A). All vectors were pseudotyped using the vesicular stomatitis virus glycoprotein (VSV-G). Initial studies to evaluate functionality of our constructs performed in a murine AM cell line (mAM) [32] revealed production and secretion of functional human AAT without major effects on cellular function by all constructs (Supplementary Figs S1 and S2 and Supplementary Table S1).

Following this initial work, we proceeded to the evaluation of our LV constructs in a primary murine MΦ population. For this purpose, lineage negative (lin^-^) cells were isolated from the bone marrow of wildtype C57BL/6J mice and transduced with the respective LV vectors at a multiplicity of infection of 10. After sorting of the transduced cells for eGFP expression, cells were differentiated toward MΦ for 10–14 days (Fig. 1B). The mean transduction efficiency in lin^-^ cells prior to sorting for 7–10 independent transductions ranged from approximately 30% for the eGFP and Cbx-EF1α-AAT construct, to ~20% for the Cbx-EFS-AAT, and only ~10% for the CAG-AAT construct (Fig. 1C). Vector copy number (VCN) in sorted and differentiated MΦ was very consistent for the individual constructs throughout the experiments with 2.15 ± 0.52 copies for the eGFP construct, 1.14 ± 0.03 copies for CAG-AAT, 2.12 ± 0.09 copies for Cbx-EF1α-AAT, and the highest number of 3.9 ± 0.24 copies per genome in Cbx-EFS-AAT MΦ (Fig. 1D). Interestingly, transduced cells showed stable expression of eGFP during the entire differentiation period of 2 weeks. At the end of differentiation, highest eGFP expression levels were observed in Cbx-EF1α-AAT MΦ followed by CAG-AAT MΦ, whereas expression from the Cbx-EFS promoter in the eGFP control as well as the Cbx-EFS-AAT construct was markedly lower (Fig. 1E, F) Similarly, the Cbx-EF1α-AAT MΦ population presented the highest expression of human *SERPINA1* mRNA, which was 2–3 times higher than the expression in CAG-AAT MΦ, while only low-level expression was detected for the Cbx-EFS-AAT construct (Fig. 1G). To assess the transgene expression on the protein level, we performed western blots on culture supernatants as well as cellular lysates of transduced MΦ. Consistently, CAG-AAT and the Cbx-EF1α-AAT MΦ secreted the highest amount of the AAT protein (Fig. 1H). Notably, the majority of the AAT was detected in the supernatant, with only a minor fraction of AAT detected in the cell lysate. Results on the protein level were further confirmed by quantification of the secreted AAT protein employing an ELISA. Here, 280 ± 177 ng/ml for CAG-AAT (*n* = 6) and 191 ± 96 ng/ml for Cbx-EF1α-AAT (*n* = 8) were measured in culture supernatants of MΦ (Fig. 1I). Again, the Cbx-EFS-AAT vector yielded only low-level expression. Interestingly, results on the protein level did not reflect the difference noted in *SERPINA1* mRNA level between the CAG-AAT and the Cbx-EF1α-AAT construct, thereby most likely reflecting differences in the efficiency of mRNA processing and/or translation. In summary, these data demonstrate efficient AAT transgene expression and secretion for the CAG-AAT as well as the Cbx-EF1α-AAT, while the Cbx-EFS-AAT only gives rise to low-level expression despite the highest detected VCN of around four among all transgene constructs.

**Fig. 2:**
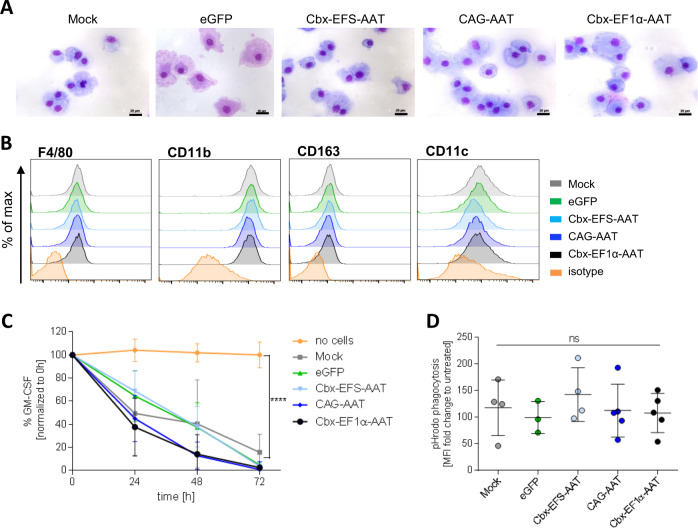
Morphology and function of murine AAT MΦ. **A** Representative May–Grünwald–Giemsa staining of MΦ cytospins. Scale bar = 20 μm. B Representative flow cytometric analysis of myeloid and MΦ-specific surface marker expression by MΦ. **C** GM-CSF uptake from cell culture medium by MΦ. A well without cells was used as a negative control. Statistical analysis was performed using two-way ANOVA with Tukey’s multiple comparisons test. eGFP, Cbx-EFS-AAT *n* = 3; mock, CAG-AAT, Cbx-EF1α-AAT *n* = 4; no cells *n* = 5. **D** Median fluorescence intensity (MFI) fold change of pHrodo *E. coli* particles after phagocytosis by murine MΦ. eGFP *n* = 3; mock, Cbx-EFS-AAT *n* = 4; CAG-AAT, Cbx-EF1α-AAT *n* = 5. All data points and *n*-numbers given in Fig. 2 are derived from independent transduction experiment. Statistical analysis was performed using one-way ANOVA with Tukey’s post-hoc test. Bars/points represent mean ± SD. ****p ≤ 0.0001; ns not significant.

Importantly, expression of the AAT transgene did not appear to induce major alterations in transduced cells. Thus, the clonogenic potential of lin^-^ hematopoietic stem and progenitor cells as assessed by the number and quality of hematopoietic colonies formed in methylcellulose medium was comparable for all constructs including the eGFP control vector (Supplementary Fig. S3A, B). Likewise, no obvious differences in MΦ differentiation were observed following AAT vector transduction. Moreover, we analyzed morphology, surface phenotype, as well as general functionality of MΦ following transduction with our AAT vectors as an additional measure to assess the safety of our approach. Here, cytospin staining revealed a homogenous morphology of mock, eGFP, and AAT vector-transduced MΦ presenting as large cells with the classical dense nucleus and large, granular cytoplasmic space (Fig. 2A). Also, AAT vector-transduced MΦ presented a typical F4/80^high^CD11b^high^CD163^+^CD11c^+^ surface marker profile comparable to control mock and eGFP MΦ (Fig. 2B). As MΦ play essential roles in inflammation, tissue homeostasis, immunological surveillance as well as a variety of other processes important for the survival of an organism, it appears crucial for our AAT MΦ to retain these properties. To this point, we evaluated cytokine uptake and phagocytotic capabilities in our studies. When the ability of MΦ to bind and internalize GM-CSF from the cell culture medium was evaluated, we observed a similar decrease in cytokine concentrations for control (mock, eGFP) and AAT vector-transduced MΦ, indicating the preservation of GM-CSF uptake capacity in AAT MΦ (Fig. 2C). Likewise, AAT MΦ generated by all three vectors effectively phagocytosed pHrodo marked *Escherichia coli* particles, similar to mock and eGFP control cells (Fig. 2D and Supplementary Fig. S3C). Presented results show that human AAT can be expressed and secreted in the context of murine MΦ without major effects on MΦ morphology, phenotype, and basic functionality.

### Functionality of transgenic human AAT

**Fig. 3:**
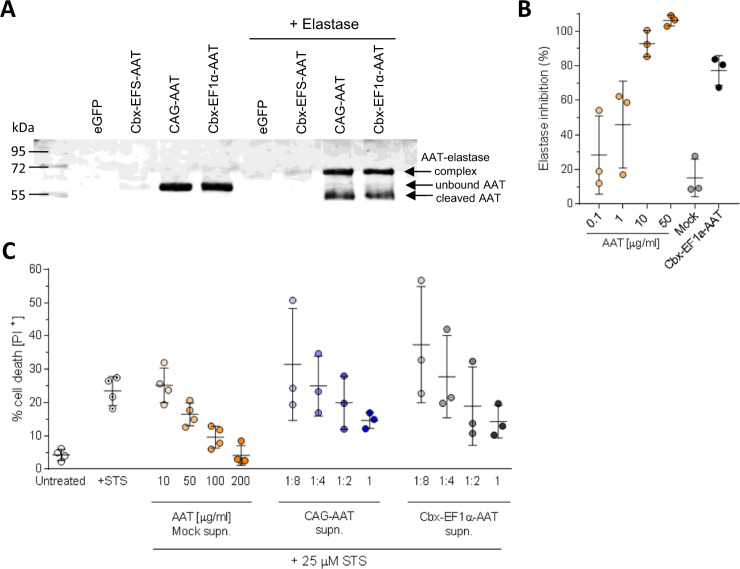
Functionality of transgenic AAT. **A** Formation of a complex between AAT and elastase. AAT present in the MΦ supernatant binds elastase and forms a complex represented as a band with higher molecular weight detected with anti-human AAT antibody in western blot analysis. In addition to the complex also a cleaved form of AAT of approx. 45–50 kD is visible. Same samples were used as for western blot in Fig. 1H. **B** Elastase inhibition by supernatant of Cbx-EF1α-AAT MΦ. AAT protein at different concentrations was used as a positive control. Supernatant of mock-transduced MΦ served as base for AAT protein and conditioned medium dilutions. *n* = 3 technical replicates. Lines represent mean ± SD. Statistical analysis was performed using one-way ANOVA with Tukey’s post-hoc test. **C** Percentage of propidium iodide (PI) positive mAM cells after apoptosis induction with 25 µM Staurosporine (STS). AAT protein at concentrations of 10–200 µg/ml in supernatant (supn.) of mock-transduced MΦ served as positive control. Supernatants of CAG-AAT and Cbx-EF1α-AAT MΦ were added pure, undiluted (1) or in 1:2, 1:4, and 1:8 dilution. Untreated, +STS, AAT 10–200 μg/ml *n* = 4; CAG-AAT and Cbx-EF1α-AAT 1:8–1 *n* = 2 biological replicates from independent transductions and 1 technical replicate. Lines represent mean ± SD.

As a next step, we investigated the functionality of the secreted AAT. The primary function of AAT, which is inhibition of proteases and in particular NE, is dependent on the cleavage of the reactive center loop in the AAT molecule by the elastase and formation of a covalent binding between the two proteins. We show that the AAT secreted by CAG-AAT and Cbx-EF1α-AAT MΦ formed a complex with elastase, visible as a higher size protein band when compared to non-elastase treated samples, proving the ability of transgenic AAT to bind elastase (Fig. 3A). Note that in addition to the complex also a cleaved form of AAT of approximately 45–50 kDa is visible. Again, AAT concentration in the supernatant of Cbx-EFS-AAT was low, and only a faint band was detectable. Next, we demonstrated the functional inhibition of PPE by AAT secreted from Cbx-EF1α-AAT MΦ, employing our established elastase assay system. Purified human protein at concentrations of 0.1–50 µg/ml, and reactions containing no AAT were used as controls. While AAT concentration of 50 µg/ml completely inhibited elastase activity, less inhibition was observed for 0.1, 1, and 10 µg/ml AAT with some variability of the elastase inhibition assay noted particularly at lower AAT concentrations. The concentrated supernatants of the Cbx-EF1α-AAT MΦ markedly inhibited the elastase reaction. The level of inhibition was comparable to 10 µg/ml AAT, thus clearly demonstrating the elastase-inhibitory function of the secreted AAT (Fig. 3B).

Since AAT represents a multifunctional protein with properties beyond elastase inhibition, such as anti-inflammatory properties, immunoregulation, and cell survival, we here evaluated the anti-apoptotic effects of secreted AAT as an additional test of protein functionality. For this purpose, mAM cells were cultured in medium conditioned by non-transduced MΦ (mock), CAG-AAT MΦ, and Cbx-EF1α-AAT MΦ and treated with 25 µM staurosporine (STS) to induce cell death. After 24 h, the percentage of propidium iodide (PI)-positive cells was measured as a marker of cell death. In control samples, mock conditioned medium supplemented with 10 µg/ml AAT protein was not able to reduce STS-induced cell death as it was comparable to the percentage of dead cells observed in the STS treatment alone (25.2 ± 4.5% vs. 23.5 ± 3.8%, *n* = 4 respectively), whereas higher concentrations of the antiprotease (50–200 µg/ml) reduced cell death in a concentration-dependent manner down to 4 ± 2.5% similar to the background levels of non-STS-treated controls (Fig. 3C). Likewise, medium conditioned by CAG-AAT and Cbx-EF1α-AAT MΦ induced substantial protection from cell death. Thus, for non-diluted supernatants only 14.6 ± 2% and 14.3 ± 4% (*n* = 3) PI^+^ cells were observed for CAG-AAT and Cbx-EF1α-AAT MΦ, respectively, while the effect decreased in a dilution-dependent manner (Fig. 3C). These results indicate a clear anti-apoptotic effect (similar to the one observed with 50 µg/ml AAT) and again demonstrate the functionality of the transgenic human AAT secreted by the murine MΦ.

### AAT MΦ engraft in the lung of *Csf2rb*^–/–^ mice and secrete human AAT in vivo

**Fig. 4:**
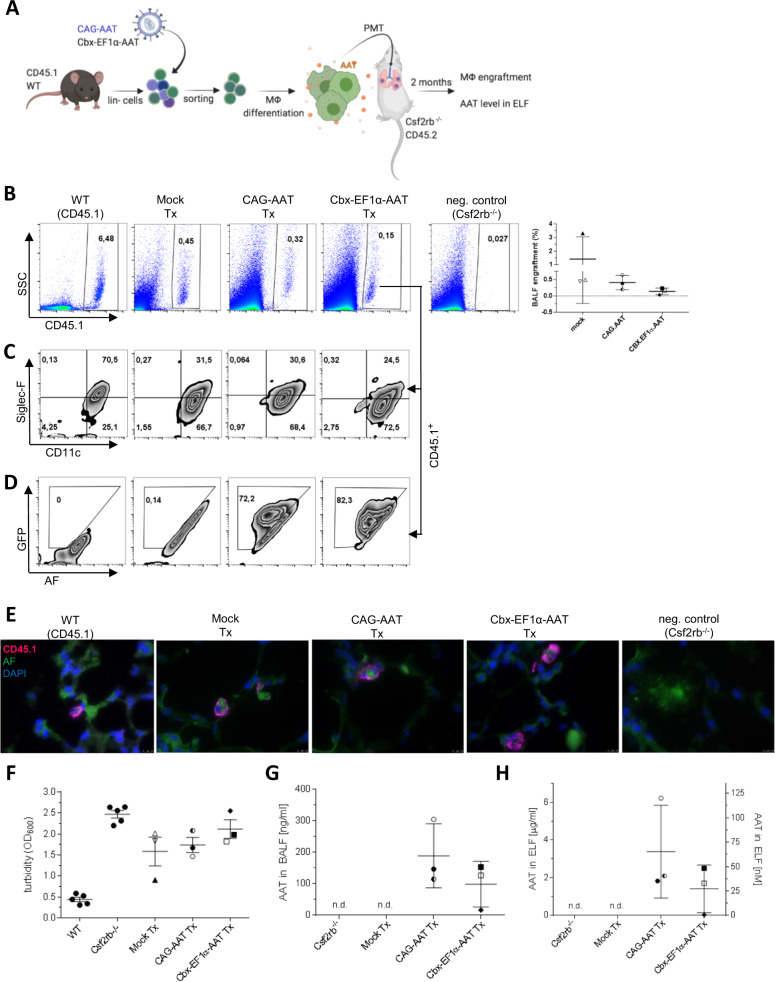
Pulmonary macrophage transplantation (PMT) of AAT MΦ into *Csf2rb*^*–/–*^ mice. **A** Schematic representation of the experimental procedures. Lineage negative (lin^-^) cells were isolated from bone marrow of CD45.1 WT mice, transduced with CAG-AAT or Cbx-EF1α-AAT vectors, sorted for eGFP^+^ cells and differentiated toward MΦ. Cells were administered intratracheally into the lungs of *Csf2rb*^*–/–*^ mice. Engraftment and AAT levels in ELF were analyzed 2 months after PMT. Created with BioRender.com. **B** Representative flow cytometry analysis of donor CD45.1^+^ cells in the BALF of recipient mice. Untreated WT (CD45.1) and *Csf2rb*^*–/–*^ mice were used as positive and negative controls, respectively. The graph represents percentage of engrafted donor MΦ in all experiments. Individual symbols for each mouse are used in all graphs. For the CAG-AAT group, all mice were transplanted with cells from completely independent lin^-^ isolations and transductions. For the Cbx-EF1α and mock groups, two animals each received cells from the same isolation and transduction and one animal received cells from an independent isolation and transduction. Expression of **C** CD11c and Siglec-F and (**D**) eGFP on CD45.1^+^ pre-gated cells. **E** Immunofluorescent staining of lung cryosections depicting the CD45.1 cells (magenta), nuclei (DAPI) and autofluorescent lung structure (green). Scale bar = 10 µm. **F** Turbidity (optical density at 600 nm, OD_600_) of BALF isolated from transplanted (mock, CAG-AAT, Cbx-EF1α-AAT), and untreated control mice (WT, *Csf2rb*^*–/–*^). **G** Quantification of human AAT in BALF by ELISA. **H** Concentration of human AAT in ELF after normalization for urea in BALF and serum. Statistical analysis was performed using one-way ANOVA with Tukey’s post-hoc test. Lines represent mean ± SD. *p ≤ 0.05; ns not significant. AF autofluorescence, BALF bronchoalveolar lavage fluid, ELF epithelial lining fluid, PMT pulmonary macrophage transplant, SSC side scatter, Tx transplanted, WT wildtype.

As Wilson et al. previously described high levels of human AAT in murine lungs following in vivo transduction of AMs [20], we here aimed to evaluate whether also AAT MΦ following intrapulmonary transplantation and engraftment may serve as a source for AAT secretion in vivo. For this purpose, we transplanted AAT MΦ into the lungs of B6;129P2-Csf2rb2^*tm1Mur*^ (*Csf2rb*^*–/–*^) mice. Due to the absence of the GM-CSF receptor and given that GM-CSF signaling is an indispensable factor for AM development and maturation, *Csf2rb*^*–/–*^ mice have an empty AM niche and allow for solid MΦ engraftment and AM differentiation following PMT [25, 26]. To mark the donor cells, we used lin^-^ cells isolated from CD45.1^+^ mice (in difference to *Csf2rb*^*–/–*^ mice harboring the CD45.2 allele) to generate our mock, CAG-AAT, or Cbx-EF1α-AAT MΦ. Two months after PMT, BALF, and lung slides, were analyzed for the presence of donor cells and human AAT (Fig. 4A). At the time point of the final analysis, flow cytometry of the BALF of transplanted mice yielded a clear population of CD45.1^+^ donor cells (Fig. 4B), demonstrating successful engraftment and persistence of murine mock, CAG-AAT and Cbx-EF1α-AAT MΦ in the recipients’ lungs for at least 2 months. As *Csf2rb*^*–/–*^ mice lack functional AMs, their lung is filled with surfactant break-down products, which are enriched in lipids and proteins and explain the background debris observed in the BALF by flow cytometry (Fig. 4B). The engrafted cells in the CAG-AAT and Cbx-EF1α-AAT cohorts expressed the AM-typical CD11c^high^Siglec-F^+^ phenotype at levels comparable to animals transplanted with mock cells, indicating that the transgenic AAT expression did not affect the upregulation of these surface markers during AM differentiation (Fig. 4C). Of note, Siglec-F expression is higher in WT CD45.1 control mice reflecting the embryonic origin of these cells [23, 33]. Importantly, the majority of CD45.1^+^ AAT MΦ were GFP^+^ indicating stable in vivo transgene expression from our lentiviral constructs (Fig. 4D). Also, donor-derived CD45.1^+^ cells not flushed into the BALF and remaining in the lung could be detected in the lung homogenate by flow cytometry (Supplementary Fig. S4A). Moreover, stained tissue sections suggest predominantly intra-alveolar localization of engrafted and persisting CD45.1^+^ donor cells 2 months after PMT (Fig. 4E and Supplementary Fig. S4B). These data again indicate efficient engraftment and in vivo AM differentiation of transplanted macrophages irrespective of AAT production and secretion. A decrease in turbidity of the BALF of *Csf2rb*^*‒/‒*^ mice can be used as an indicator for the presence of functional AMs [26, 27]. In this respect, BALF turbidity of *Csf2rb*^*–/–*^ mice receiving mock or CAG-AAT MΦ was markedly lower when compared to not transplanted *Csf2rb*^*–/–*^ mice. Similar data were obtained for Cbx-EF1α-AAT MΦ in two of the three analyzed animals, while the mouse with the lowest engraftment did not show improvement of turbidity (Fig. 4F). Overall these results indicate the degradation of the accumulated surfactant material, proper AM differentiation, and the functionality of transplanted cells in five out of six animals across the two experimental groups. These results clearly indicate the degradation of the accumulated surfactant material, proper AM differentiation, and the functionality of transplanted cells. In addition, CAG-AAT and Cbx-EF1α-AAT MΦ were able to secrete the human AAT into the lung of transplanted mice resulting in mean AAT BALF levels of 183 ng/mL for the CAG-AAT and 99 ng/mL for the Cbx-EF1α-AAT group, respectively (Fig. 4G). After urea-based correction for the dilution of the BALF during flushing, this correlates to AAT concentration in the ELF ranging from 2 to 6 μg/ml (35–125 nM) (Fig. 4H). Taken together, the data show that murine AAT MΦ can engraft in the lung and develop into functional AMs with an efficiency similar to mock MΦ. AAT MΦ can also secrete human AAT in vivo into the lungs, though the levels obtained fall short of the 1.2 µM ELF threshold postulated to confer protection from AATD-associated emphysema development [8, 17].

### AAT overexpression in human cells

After proving the secretion of functional human AAT by murine MΦ in vitro and in vivo, as the next step toward clinical translation, we evaluate the transgenic production of AAT in human myeloid cells. First studies were performed in the myeloid U937 cell line and we demonstrated effective secretion of AAT following transduction with the CAG-AAT LV vectors, which was previously confirmed to induce the strongest AAT secretion in this model (Supplementary Fig. S5A, B). Importantly, the production of the antiprotease did not affect the basic characteristics of U937 cells, including the proliferation rate (Supplementary Fig. S5C).

**Fig. 5:**
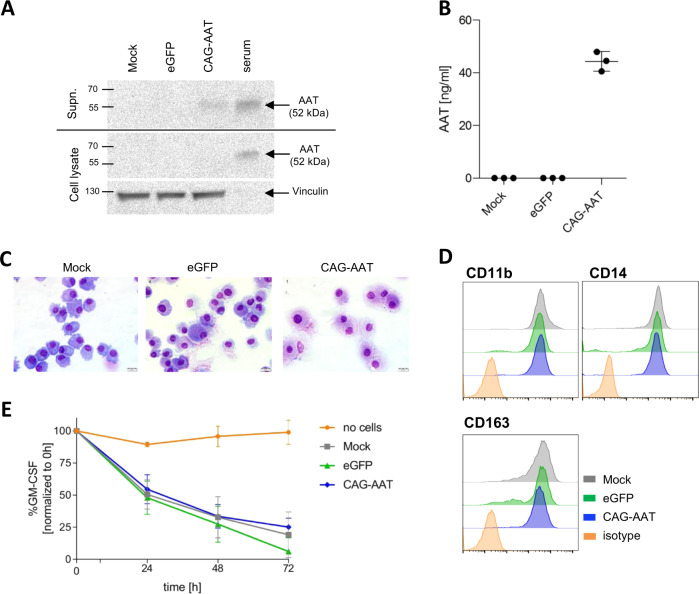
Expression of AAT in human MΦ. **A** Representative AAT western blot analysis of human MΦ supernatant and MΦ lysates. Human serum was used as a positive control. Vinculin band in cellular lysates served as loading control. **B** ELISA quantification of AAT secretion by human MΦ. *n* = 3. **C** Representative May–Grünwald–Giemsa staining of MΦ cytospins. Scale bar = 20 μm. **D** Representative flow cytometric analysis of myeloid and MΦ-specific surface marker expression by human MΦ. **E** GM-CSF uptake from cell culture medium by MΦ. A well without cells was used as a negative control. *n* = 3. All data represent independent cell isolations and transductions.

In the next step, we transduced human cord blood CD34^+^ hematopoietic stem/progenitor cells with the CAG-AAT construct, sorted for transduced cells by GFP expression, and differentiated these cells into MΦ. CAG-AAT MΦ were able to successfully secrete transgenic AAT into the supernatant as demonstrated by western blot and ELISA (Fig. 5A, B), although levels of secreted AAT were markedly lower than for murine MΦ. Again, morphology, expression of MΦ-typical surface markers CD11b, CD14, and CD163, as well as basic functionality measured as GM-CSF uptake capacity were not affected by transgenic AAT expression (Fig. 5C–E), indicating that human MΦ can also be engineered to secrete the AAT transgene without adverse effects on basic cell characteristics and functionality.

## Discussion

Despite the introduction of substitution therapy with purified serum AAT protein, early-onset emphysema still represents a considerable health issue in AATD patients, particularly in smokers. In part, these problems are related to the substantial costs of substitution therapy of more than 100,000 $/year or poor compliance of the patients, but even in optimal settings not all patients profit from substitution therapy, and an impact on overall survival is difficult to demonstrate [34]. Thus, new therapeutic options, including genetic approaches, are currently under investigation. Such gene-therapeutic strategies primarily employ AAV- or lentivirus-based delivery systems to target muscle cells [15], hepatocytes [35], or directly the lung [20, 21]. In the clinical setting, however, all these approaches have failed to reach therapeutic AAT levels in the serum and/or the pulmonary ELF, so far.

The approach presented here is based on the promising results reported following in vivo lentiviral transduction of AM in murine models [20] as well as the recent demonstration of successful PMT in the context of congenital lung diseases such as pulmonary alveolar proteinosis in murine proof-of-principle studies [25, 26]. To obtain optimal levels of AAT secretion from MΦ, we investigated three different promoter constructs. Both, the short (EFS) and long (EF1α) version of the elongation factor 1-alpha promoter already have proven safe and effective in clinical trials to express therapeutic transgenes in the hematopoietic system including myeloid cells [36, 37]. To increase transgene expression and prevent silencing, we combined these promoters with the Cbx3 element derived from the 5’ part of the HNRPA2B1/CBX3 ubiquitous chromatin opening element, a well-known epigenetic regulatory sequence containing long stretches of methylation resistant CpG residues [38]. The Cbx3 element repeatedly has been shown to stabilize transgene expression in cell lines and primary cells, including monocytes/macrophages [30, 39]. Also, the third promoter construct the hybrid CAG promoter repeatedly has been demonstrated to direct strong, ubiquitous, and silencing resistant transgene expression in the context of lentiviral as well as AAV vectors and already has been applied in clinical settings [14, 31, 40]. Moreover, the CAG promoter has been shown to induce robust transgene expression in myeloid cells lines and in vitro generated macrophages [41].

In our hands, efficient secretion of human AAT by primary murine AAT MΦ was obtained with the CAG as well as the Cbx-EF1α promoter construct and AAT levels detectable after 24 h ranged from 100 to 600 ng/ml. While this was similar to levels detected in murine mAM cells, much higher secretion levels have been reported in cell line models by other groups. Thus, AAT levels of 60 and 10 µg/ml, respectively, were observed for the CAG promoter in the J774A.1 cells or the CMV promoter in HEK293T cells [42, 43]. However, direct comparison of levels between different studies is difficult, as important parameters required for standardization such as number of cells seeded, number of genomic integrations, the volume of culture medium, or time of sample collection vary considerably. In addition, in an initial set of experiments we could demonstrate AAT production and secretion from human MΦ with our vector constructs without major effects on phenotype or basic functionality, although AAT levels in these initial experiments were markedly lower than in murine cells.

Following endotracheal transplantation, transgenic AAT MΦ engrafted long term into the pulmonary microenvironment and differentiated toward a CD11c^high^Siglec-F^+^ AM phenotype with a similar efficacy as mock controls. Siglec-F levels in AMs of the CAG-AAT and Cbx-EF1α-AAT-transplanted cohorts were comparable to animals transplanted with mock cells. However, Siglec-F expression in cells recovered after transplantation was lower than in AMs of wildtype controls reflecting the embryonic origin of AMs in wildtype animals. Moreover, animals in both groups, CAG-AAT and Cbx-EF1α-AAT, demonstrated marked improvement in BALF protein levels, although for the Cbx-EF1α-AAT cohort this finding was restricted to two out of three animals. Of note, the animal with lack of improvement in BALF protein levels also failed to produce any meaningful BALF or ELF AAT levels. Intra-alveolar protein degradation clearly appeared to be correlated with engraftment levels and the two outliers in Fig. 4F in the Cbx-EF1α-AAT as well as the mock cohort represented animals with particularly low or high engraftment, respectively. Thus, our data seem to indicate that transgenic AAT expression per se does not have a major impact on cell fate and functionality following intratracheal transplantation, even though these results have to be interpreted with caution given the small cohort size.

An important concept in intrapulmonary gene and cell therapy approaches is the notion that AAT levels far below serum levels may protect from AATD-associated emphysema formation. Normal AAT plasma levels are 1–2 mg/ml in humans [4] and 3–4 mg/ml in mice with the higher levels in mice representing the presence of up to five *Serpina* paralog genes in the murine genome. However, physiologically only 5–10% of AAT serum levels are detected in the ELF as the tight junctions of the alveolar epithelium restrict the diffusion of AAT to the intra-alveolar space. Based on observational studies of AATD patients, a threshold serum level of 11 µM, or an ELF level of at least 1.2 µM (equal to 63 µg/ml) AAT has been postulated for clinically effective AAT substitution therapy to protect from emphysema formation. [8, 44, 45].

In our studies only 2–6 μg/ml of AAT were detected in the ELF falling well short of the 1.2 µM threshold in human (63 µg/ml) or physiological level in mouse (150–400 µg/ml) [8, 45]. On this background substantial improvements in vector design and engraftment efficiency appear warranted to improve AAT levels [46–48]. However, it is also possible that eventually protective levels in the ELF turn out substantially lower than the anticipated threshold. Thus, meaningful studies in relevant in vivo models are needed.

In the past, studies to assess the efficacy of AATD-directed therapies in animal models have been problematic, as murine models based on cigarette smoke exposure or elastase instillation only poorly reflected the lung phenotype of AATD [49]. Recently, however, a bona fide murine AATD model has been developed by a quintuple knockout of the murine *Serpina1a-e* genes [50]. These mice develop spontaneous emphysema at the age of 1 year and emphysema formation can be accelerated by chronic exposure to inflammatory stimuli. Thus, the model can be expected to represent a valuable tool to validate novel therapeutic approaches to AATD. A potential caveat of studying macrophage transplantation in the AATD model, however, is represented by the fact that in this, as well as the vast majority of other murine models, an intact population of AMs is present and the occupied lung niche may prevent the efficient engraftment of the transplanted AAT MΦ. Therefore, pre-conditioning of the lung to remove AMs and open the niche for delivered AAT MΦ may be necessary. In this context, clodronate liposomes have already been demonstrated to deplete distinct populations of MΦ, including AM [46, 47], and may be employed in future studies.

Thus, in summary we here report the in vitro generation of AAT MΦ, which can engraft into the pulmonary microenvironment and convert into an AM phenotype. Transgenic human AAT was detected in the lung ELF of transplanted animals. However, levels are clearly lower than the 1.2 µM threshold currently postulated as necessary for therapeutic benefit and need to be improved before the concept can be further tested in relevant murine models such as the AATD mouse described above.

## Materials and methods

### Cultivation of cell lines

The mAM cell line was provided by Takuji Suzuki and Bruce Trapnell from Cincinatti Childrens Hospital Medical Center [51]. Cells were cultured in DMEM medium (Gibco, Life Technologies, Paisley, UK) supplemented with 10% FBS superior (Millipore, Billerica, MA, USA), 100 U/ml penicillin/streptomycin (P/S, Thermo Fisher Scientific, Waltham, MA, USA), and 20 mM HEPES (Thermo Fisher Scientific). Cell line was regularly tested for mycoplasma contamination. For lentiviral transduction, 4 μg/ml protamine sulfate was added to the culture medium. U937 cells were cultured in RPMI1640 (Gibco) supplemented with 10% FBS superior (Millipore) and 100 U/ml P/S. Both cell lines were cultured under standard conditions at 37 °C and 5% CO_2_ and passaged every 3–4 days. Each cell line was only transduced once and samples for the assays were taken at different time points during continuous culture of the lines.

### Production and titration of lentiviral vectors

VSV-G pseudotyped third-generation SIN LV were produced in HEK293T cell line and titrated on SC-1 cells as previously described [51]. Briefly, HEK293T cells were transfected with the pcDNA3.GP.4xCTE (encoding HIV-1 gag/pol), pRSV-Rev, pMD.G (encoding the envelope VSV-G), and the desired lentiviral transgene plasmid. After 24 and 48 h the supernatant was filtered through 0.22 µm filter and centrifuged overnight at 10,000×g, 4 °C. The pellet was resuspended in StemSpan medium (Stem Cell Technologies, Vancouver, Canada) and kept at −80 °C. The average titers of the vector preparations obtained for the different constructs were 1.58 ± 0.67  × 10^8^ for Cbx-EFS-GFP, 1.62 ± 1.13 × 10^8^ for Cbx-EFS-AAT, 2.03 ± 2.2 × 10^8^ for Cbx-EF1α-AAT, and 0.4 ± 0.3 × 10^8^ for CAG-AAT.

### Vector copy number (VCN) quantification

The VCN was quantified as previously described [52]. Briefly, genomic DNA was isolated using the GenElute Mammalian Genomic DNA Miniprep Kit (Sigma-Aldrich, St. Louis, MO, USA) according to the manufacturer’s instructions. The VCN was determined using the Taq-Man based qRT-PCR on StepOne Plus thermocycler (Applied Biosystems, Foster City, CA, USA) targeting the wPRE sequence of the LV vector and the PTPB2 as a house-keeping gene.

### Murine lin^-^ cell isolation, transduction, and cultivation

Lineage negative (lin^-^) cells were isolated from the bone marrow of C57BL/6J, C57BL/6J*HanZtm* and B6.SJL-*Ptprc*^*a*^*-Pep3*^*b*^/BoyJZtm (Ly5.1*;* CD45.1) mice using the lineage cell depletion kit mouse (Miltenyi Biotech, Bergisch-Gladbach, Germany) according to the manufacturer’s instructions. Lin^-^ cells were cultured in StemSpan medium (Stem Cell Technologies) supplemented with 100 U/ml P/S, 2 mM L-glutamine (Thermo Fisher Scientific), 10 ng/ml SCF, 20 ng/ml TPO, 20 ng/ml IGF-2, and 10 ng/ml FGF-1 (STIF, all Peprotech, Rocky Hill, NJ, USA). The next day, lin^-^ cells were transduced with LV vectors at MOI 10 on RetroNectin-coated plates (Takara Bio, Inc., Shiga, Japan) as recommended by the manufacturer. Four days after the transduction, the STIF medium was supplemented with 10 ng/ml IL-3. Cells were sorted for GFP^+^ cells and transferred to RPMI1640 (Gibco) supplemented with 10% FCS, 100 U/ml P/S, and 30% of conditioned medium from the M-CSF producing L929 cell line. The differentiation medium was supplemented with IL-3 for the first 2 days. Then, 10–14 days after sorting, differentiated MΦ were used for in vitro assays and in vivo experiments. The number of cells that was generated with a single transduction was varying and thus, not all assays could be performed with one cell batch.

### Cord blood CD34+ cell isolation, transduction, and cultivation

Umbilical cord blood from donors who gave informed consent was provided from Hannover Medical School. Experiments were approved by the local ethic committee (approval numbers: 1303-2012). CD34+ cells were isolated as previously described [51]. Briefly, after gradient centrifugation, stem and progenitor cells were enriched by magnetic sorting using the CD34 MicroBead Kit (Miltenyi). Cells were cultured in StemSpan (Stem Cell Technologies) supplemented with human cytokines (SCF, Flt3, TPO, all Peprotech), and transduced at the MOI 15 on RetroNectin-coated plates (Takara) as recommended by the manufacturer. Sorted GFP+ cells were differentiated toward MΦ in RPMI1640 containing 10% FCS, human M-CSF, GM-CSF and IL-3 (all Peprotech).

### AAT sandwich ELISA

To prepare the supernatants, cells were seeded at defined densities (1 × 10^5^ for mAM cells, 5 × 10^5^ for primary murine MΦ) on 12-well tissue culture plates in 0.5 ml of respective cell culture medium. Supernatants were collected after 24 h (mAM and MΦ), 48 h, and 72 h (mAM) and stored at −80 °C until the day of analysis.

High binding 96-well plates (Sarstedt, Nuembrecht, Germany) were coated with 100 μl of 5 μg/ml rabbit anti-human AAT monoclonal antibody (Cat. no. A0409; Sigma-Aldrich) in carbonate-bicarbonate buffer (Medicago, Quebec City, Canada) o/n at 4 °C. Wells were washed four times with 200 μl PBS + 0.05% Tween20 (AppliChem, Darmstadt, Germany) and blocked for 1 h with 1% BSA (Carl ROTH, Karlsruhe, Germany) in PBS at RT. Standard AAT (Calbiochem, San Diego, CA, USA) was prepared in a range of 3–800 ng/ml by serial dilutions in blocking solution. All samples were run in the assay in duplicates. Then, 100 µl of standard and test samples was added to the wells and incubated for 2 h at RT. Wells were washed four times and the secondary, HRP-conjugated mouse anti-human AAT antibody was added (1:5000 in blocking solution, Novus Biologicals, Littleton, CO, USA, Cat. no. NBP1-05147H) for 1 h at RT. Wells were washed four times again and 100 μl of TMB solution (Abcam, Cambridge, UK) was added. To stop the reaction, 50 μl of 2 N H_2_SO_4_ (Carl ROTH) was added and the result was immediately analyzed at 405 nm on a Model 680 microplate reader (Bio-Rad, Hercules, CA, USA).

### Phagocytosis assay

One day before the assay, cells were seeded at a density of 1 × 10^5^ on a 12-well tissue culture plate. The following day, 10 μl of pHrodo red *E. coli* bio-particles (Invitrogen, Carlsbad, CA, USA) were added and the cells were incubated for 1.5 h at 37 °C. For each condition, one well without pHrodo particles served as a negative control. After the incubation time, fluorescent pictures were taken and the cells were collected for flow cytometry analysis.

### GM-CSF clearance assay

Cells were seeded at a density of 1 × 10^5^ on a 24-well tissue culture plate. On the next day, the medium was changed to 1 ml X-Vivo 15 (Lonza, Basel, Switzerland) containing 1 ng/ml GM-CSF and a 100 μl aliquot was taken as a time point 0 h. The next samples were taken at 24, 48, and 72 h and stored at −20 °C until analysis. The level of GM-CSF was assessed with the Ready-Set-Go!® ELISA Kit (eBioscience, San Diego, CA, USA) according to the manufacturer’s instructions. Supernatant from a well without cells was used as a negative control. The result was normalized to the time point 0 h.

### Western blot

For the preparation of mAM cell and murine MΦ culture supernatants, 2.5 × 10^5^ or 5 × 10^5^ cells were seeded on 12-well tissue culture plates. The next day, the medium was changed to 0.5 ml without FCS (DMEM with 100 U/ml P/S for mAM cells or RPMI with 100 U/ml P/S and 50 ng/ml murine M-CSF for primary MΦ). After 24 h, the supernatant was collected and stored at −80 °C until further analysis. Cellular proteins were isolated in RIPA buffer (Sigma-Aldrich) containing proteinase inhibitor (cOmplete™ Protease Inhibitor cocktail; Sigma-Aldrich). The protein concentration was determined using the Pierce BCA Protein Assay Kit (Thermo Fisher Scientific) according to the manufacturer’s instructions.

For western blot analysis, 35 μl of the supernatant or 30 μg of cell lysate proteins was loaded onto 12% acrylamide gels. After blotting the proteins to PVDF membranes, they were incubated with the primary anti-AAT (Invitrogen, Cat. no. MA5-15521) or anti-vinculin (Sigma-Aldrich, Cat. no. V9131) antibodies in 5% milk o/n at 4 °C. After washing, membranes were incubated with the secondary anti-mouse HRP-conjugated antibody (Jackson ImmunoResearch, West Grove, PA, USA) for 45 min at RT. The signal was detected using the SuperSignal^TM^ West Pico or Femto Chemiluminescent Substrate (Thermo Fisher Scientific) according to the manufacturer’s instruction on a ChemiDoc XRS+ (Bio-Rad).

### Elastase inhibition assay

mAM cells were seeded at a density of 4 × 10^5^ on a 6-well tissue culture plate. The next day, 2 ml of medium without FBS and phenol red was added (DMEM FluoroBrite + 100 U/ml P/S + 2 mM L-glutamine). For the preparation of the primary MΦ supernatant, 1 × 10^6^ cells were seeded on 6-well tissue culture plates. The next day, 1.5 ml of medium without FBS was added (DMEM FluoroBrite + 50 ng/ml muM-CSF + 100 U/ml P/S + 2mM L-glutamine). After 48 h, the supernatant was collected and concentrated around ten times with 10 kDa Amicon® Ultra 2 ml Centrifugal Filters (Merck, Darmstadt, Germany) according to the manufacturer’s instructions and kept at −80 °C until analysis.

Ten-microliter supernatant concentrate or the AAT protein (Zemaira from CSL Behring, King of Prussia, PA, USA) was incubated in 96-well plates in duplicates with 5 μl of 20 μg/ml PPE (Sigma-Aldrich) and 35 μl of 0.1 M, Tris buffer (pH 8.0) at 37 °C for 20 min. Subsequently, 225 μl Tris buffer and 25 μl of 1 mg/ml PPE substrate (N-Succinyl-Ala-Ala-Ala-p-nitroanilide, Sigma-Aldrich) were added. Blank (without PPE) and an uninhibited reaction (without AAT) were run as controls in concentrated cell culture medium. The OD405 was measured on an InfiniteM200 plate reader (Tecan, Maennedorf, Switzerland) at 37 °C every 30 s for 10 min.

For the determination of the elastase activity, the OD405 value of the blank sample was subtracted, and a slope of the reaction was calculated. Subsequently, the following equation was used to calculate the percentage of elastase inhibition:

$$\% \;{{elsastase}}\;{{inhibition}} = \frac{{{\mathrm{slope}}\;({\mathrm{uninhibited}}\;{\mathrm{reaction}}) - {\mathrm{slope}}\;({\mathrm{test}}\;{\mathrm{sample}})}}{{{\mathrm{slope}}\;{\mathrm{uninhibited}}\;{\mathrm{reaction}}}} \ast 100\% $$

### Elastase-AAT complex formation

Ten-microliter supernatant from each condition was incubated with equal volumes of PBS or elastase (Sigma-Aldrich) at a final concentration of 5 ng/ml for 1 h at 37 °C. The reaction was terminated by adding equal volumes of 2X SDS sample buffer and samples were incubated at 95 °C for 5 min. Sample mixtures were shortly spun down and 20 μl were loaded and separated by SDS-PAGE on 10% gels prior to the transfer to a PVDF membrane. Membranes were blocked for 1 h with TBS + 0.01% tween containing 5% BSA (Millipore) followed by overnight incubation at 4 °C with the primary polyclonal rabbit anti-human AAT antibody (Cat. no. A0012, DAKO, Glostrup, Denmark). AAT and elastase complexes were visualized with HRP-conjugated polyclonal anti-rabbit antibody (DAKO) and enhanced by ECL western blotting substrate (Bio-Rad). Images were taken using Chemidoc Touch imaging system (Bio-Rad).

### STS-induced apoptosis

Supernatants from culture of murine MΦ were collected and kept at −80 °C. mAM mock cells were seeded at a density of 2 × 10^4^ on 24-well tissue culture plates. The next day, MΦ supernatants were thawed and warmed to 37 °C. The mAM culture medium was aspirated and 330 µl of the respective mock, CAG-AAT or Cbx-EF1α-AAT supernatant medium was added at different dilutions (1–1:8 dilution). As a control, human AAT protein (Zemaira, CSL Behring) at different concentrations was added to the mock supernatant. Cells were incubated at 37 °C for 2 h. Subsequently, STS was added at a final concentration of 25 µM. One well of cells with the mock supernatant without AAT served as a positive control for the apoptosis, a well without AAT and STS as a negative control. After 24 h, cells were trypsinized, stained with 1 µl PI (eBioscience) and analyzed on a CytoFLEX S (Beckman Coulter, Brea, CA, USA). The analysis was performed using the FlowJo v.10 software (Becton Dickinson, Franklin Lakes, NJ, USA).

### Flow cytometry

Trypsinized cells or BALF and lung samples were resuspended in 100 μl FACS buffer (PBS with 2% FCS and 1% EDTA), stained with 1 μl antibody and incubated for 45 min at 4 °C (anti-mouse antibodies: CD11c APC (Cat. no. 17-0114-81), CD45.1 PE-Cy7 (Cat. no. 25-0453-81), CD163 PE-Cy7 (Cat. no. 12-1631-82), F4/80 APC (Cat. no. 17-4801-80), CD11b PE (Cat. no. 12-0112-82)—all from eBioscience, Siglec-F from BDBioscience (Cat. no. 552126); anti-human antibodies: CD11b APC from eBioscience (Cat. no. 17-0118-41), CD14 PE (Cat. no. 12-0116-41), and CD163 APC from Invitrogen (Cat. no. 17-1639-41)). After washing with PBS, samples were measured on a CytoFLEX S (Beckman Coulter). The analysis was performed using the FlowJo v.10 software (Becton Dickinson).

### Cytospin

A total of 20,000–40,000 cells in 150 μl PBS were spun onto a glass slide at 700×g for 10 min in a Cytofuge (Medite, Burgdorf, Germany). After allowing the slides to air-dry the slides were incubated in the May–Grünwald solution for 5 min, washed in deionized water, and incubated in Giemsa solution (1:20 in water). After washing the slides with water, they were left to dry and mounted in ROTI™ mounting medium (Carl ROTH).

### Clonogenic assay

After sorting of the lin^-^ cells, 1.5 × 10^3^ cells/ml were cultured in mouse methylcellulose complete media (R&D systems, Minneapolis, MN, USA) on 8.8 cm^2^ cell culture dishes with a 2 mm grid (Thermo Fisher Scientific) and kept in a wet chamber in a cell culture incubator. The number of colonies composed of more than 50 cells was counted 10–14 days after seeding.

### qRT-PCR

Total RNA was extracted from cells using TRIsure (Bioline, London, UK) according to the manufacturer’s instructions. Then, 1  μg of RNA was treated with the DNAse I (Thermo Fisher Scientific) and reverse transcribed using Oligo (dT) primers with the RevertAid H Minus Reverse Transcriptase kit (Thermo Fisher Scientific) according to the manufacturer’s instructions. For the qRT-PCR reaction, 1 μl of the cDNA was mixed with 7.5 μl of SybrGreen PCR master mix (Applied Biosystems), 1 μl of the primer mix for human *SERPINA1* (Integrated DNA Technologies, Coralville, IA, USA) or murine *Actb* (Qiagen, Hilden, Germany) and 5.5 μl H_2_O. PCR reaction was run in duplicates on a StepOne Plus thermocycler (Applied Biosystems) for 40 cycles at 95 °C for 15 s and 60 °C for 1 min.

### Protein preparation, in-gel digest, and mass spectrometry (MS)

Proteins from cell culture supernatants of CAG-AAT mAM cells were precipitated using the the TCA-NLS method [52]. Briefly, 10% N-Lauroylsarcosine was added to the supernatant to a final concentration of 0.1% and mixed. In the next step, 100% TCA was added to a final concentration of 7.5%, mixed and precipitated on ice in dark for at least 2 h. The solution was centrifuged at 10,000×g for 15 min at 4 °C and the pellet was then washed with 1 ml tetrahydrofurane. After centrifuging the sample at the maximum speed for 10 min at 4 °C, the pellet was dried on ice for 30 min and resuspended in sample buffer (30 mM Tris-HCl (pH 8.0), 7 M urea (Carl ROTH), 2 M thiourea (Sigma-Aldrich), and 4% CHAPS (Carl ROTH)).

Samples were separated by SDS-PAGE on 12% gels and stained with InstantBlue protein stain (Novus Biologicals). Bands at the expected molecular weight of AAT (~52 kDa) were excised and cut into small pieces. The gel pieces were then subjected to in-gel digestion with first PNGaseF for liberation of N-glycans and second with trypsin for generation of peptides. Briefly, the gel pieces were dehydrated with acetonitrile (ACN, Merck), proteins in the gel were reduced with 10 mM ditihothreitol (Sigma-Aldrich) in 100 mM ammonium bicarbonate buffer (AmBic) and subsequently carbamidomethylated with 100 mM iodoacetamide (Sigma-Aldrich) in 100 mM AmBic. After dehydrating with ACN, rehydrated with 50 mM AmBic, and dehydrating with ACN, peptide-N-glycosidase F (PNGaseF from *Elizabethkingia meningoseptica*; BioReagent grade, Sigma-Aldrich) was added at a final concentration of 0.1 U/µl and incubated o/n at 37 °C. The supernatant containing the N-glycans was dried in a vacuum centrifuge. Subsequently, the same gel pieces were dehydrated with ACN, rehydrated with 50 mM AmBic containing 20 ng/µl sequencing-grade trypsin (Promega, Madison, WI, USA) and incubated for 16 h at 37 °C. The peptides were extracted using ACN and dried in a vacuum centrifuge. In the next step, dried peptides were dissolved in 15 µl 2% ACN and 0.1% formic acid and the supernatant was subjected to LC-MS/MS analysis. Reverse phase chromatography using acetonitrile as an eluent was performed on a nanoACQUITY UPLC (Waters, Milford, MA, USA) device equipped with an analytical column (Waters, BEH130 C18, 100 µm × 100 mm, 1.7 µm particle size) coupled online to an ESI Q-TOF Ultima (Waters). Spectra were recorded in positive reflection mode and peptides were automatically subjected to fragmentation. ProteinLynxTM Global Server software V2.1 (Waters) was used to identify proteins by searching against human database. Carbamidomethylation was set as fixed modification and oxidation of methionine as variable modification. Up to one missed cleavage was allowed. Peptide tolerance was set to 100 ppm and fragment tolerance to 0.1 Da and the validation filter was selected in the ProteinLynxTM Global Server software.

### Multiplexed capillary gel electrophoresis coupled to laser-induced fluorescence detection (xCGE-LIF)

Extracted N-glycans were labeled with 8-aminopyrene-1,3,6-trisulfonic acid (APTS, Sigma-Aldrich) and excess APTS was removed by hydrophilic interaction liquid chromatography. Labeled glycans were separated and monitored by xCGE-LIF applying a remodeld ABI PRISM® 3100-*Avant* Genetic Analyser (Thermo Fisher Scientific). xCGE-LIF data were assessed and processed using GeneMapperTM Software v.3.7 [53]. We determined the relative intensity of all N-glycan peaks by calculating the relative signal height of an individual peak in relation to the sum of signal intensities of all peaks.

### In vivo experiments

C57BL/6J, C57BL/6J*HanZtm*, and B6.SJL-*Ptprc*^*a*^*-Pep3*^*b*^/BoyJZtm (CD45.1) mice were used for isolation of bone marrow lin^-^ cells. B6;129P2-Csf2rb2^*tm1Mur*^ (*Csf2rb*^*–/–*^) mice at the age of 15–21 weeks were used as recipients of the MΦ transplantation. Mice were obtained from and housed in the central animal facility of Hannover Medical School where they were kept in specific-pathogen-free or individually ventilated cage conditions. All in vivo experiments were approved by the Lower Saxony State animal welfare authorities.

Terminally differentiated MΦ derived from CD45.1 lin^-^ cells were harvested and 2.5–4 × 10^6^ MΦ were resuspended in 50 μl PBS. *Csf2rb*^*–/–*^ mice were anesthetized, a 22G Introcan Safety^®^ IV catheter (B. Braun, Melsungen, Germany) without the sharp end of the needle was inserted into the trachea and the cell solution was applied into the catheter. The delivery of the cells into the lung was confirmed by the quick inhalation of the solution by the mouse.

### Analysis of in vivo experiments

Two months after PMT, mice were sacrificed with an overdose of Ketamine (10 mg per mouse) and Rompun (1 mg per mouse) by intraperitoneal injection.

To obtain the BALF, the trachea was cannulated with a 20 G Introcan Safety® IV catheter (B. Braun). The left lung was pinched off and the right lung lobes were flushed twice with PBS (600 + 500 μl–three times in and out). BALF was centrifuged to pellet the cells and the supernatant was aliquoted and stored at –80 °C for further analysis. The cells were distributed into 5 ml round-bottom tubes for staining. The left lung lobe was filled with the Tissue-Tek OCT compound solution (Sakura Finetek, Torrance, CA, USA), and kept at –80 °C until further processing.

Blood was collected via cannulation of the inferior vena cava and left at room temperature for 20 min to clot. After 10 min centrifugation at 1800×g, 4 °C, the serum phase was collected, aliquoted, and stored at –80 °C.

### Immunofluorescence analysis of lung slices

Left lung lobes frozen after filling them with Tissue-Tek OCT compound solution were cryosectioned into 6 µm slices and fixed and permeabilized in ice-cold acetone for 10 min. Dried slides were rehydrated in PBS for 10 min and subsequently blocked with the 10% swine serum in PBS for 30 min at room temperature. After removing the blocking solution, staining with the APC-conjugated CD45.1 antibody (eBioscience, Cat. no. 17-0453-82) was performed at dilution of 1:100 in the blocking solution and incubated o/n at 4 °C in a wet chamber. Following washing, nuclei were stained with DAPI (1:10000). The slides were covered in the mounting medium (DAKO). Pictures of the lung sections were taken with a fluorescent microscope Leica DM6000B (Leica Microsystems).

### Urea quantification in serum and BALF

To calculate the AAT concentration in ELF, the dilution of the ELF with PBS was determined by measuring the urea concentration in serum and BALF with the QuantiChrom™ Urea Assay Kit (BioAssay Systems, Hayward, CA, USA) following the manufacturer’s instructions. The fold difference in the concentration in serum and BALF represents the dilution factor.

### Statistics

GraphPad Prism version 8 (Graphpad Software, San Diego, CA, USA) was used to perform one-way or two-way ANOVA. If not stated otherwise, data are represented as mean plus/minus standard deviation.

## Supplementary information

Supplemental material
