# Supplementary material for: Pulmonary transplantation of alpha-1 antitrypsin (AAT)-transgenic macrophages provides a source of functional human AAT in vivo
Source: Gene Ther. 2021 Jul 19;28(9):477–93. doi: 10.1038/s41434-021-00269-3 (PMC8455329; doi:10.1038/s41434-021-00269-3)
Supplement: Supplementary file 1 — Supplemental material [file 41434_2021_269_MOESM1_ESM.docx]

**
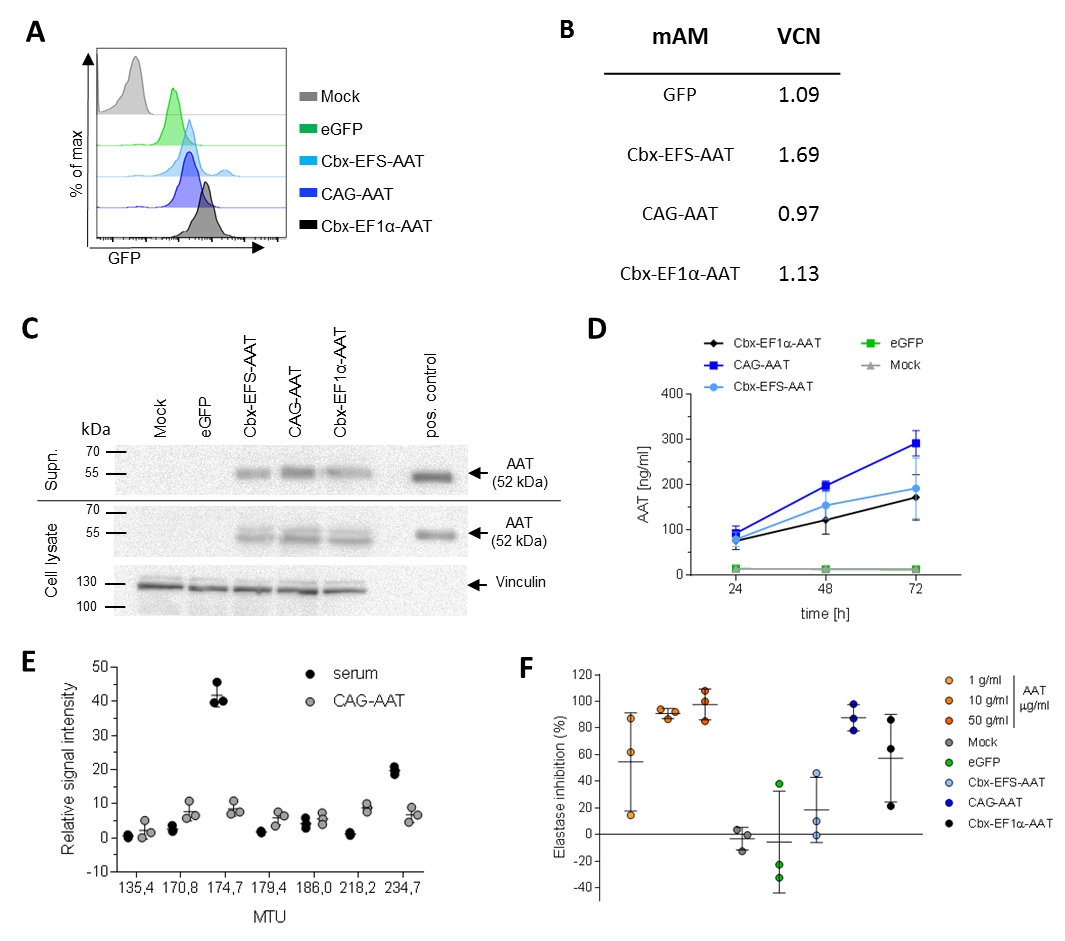
**

**Supplementary Fig. 1. Overexpression of human AAT in the murine mAM cell line.** The cell line was only generated once in a single transduction. Replications were performed by taking samples at different time points during continuous culture and thus, represent one biological replicate and n numbers refer to number of replicates at different time points of the continuous culture. Therefore, no statistical analysis was performed. (A) Representative histograms of the eGFP expression in transduced and sorted mAM cells. (B) Vector copy number (VCN) determined in transduced mAM cells. (C) Representative Western blot analysis of cell supernatant and cellular lysates for presence of human AAT. Human serum was used as a positive control. Vinculin band in cellular lysates serves as a loading control. (D) ELISA quantification of human AAT in supernatant of mAM cells after 24h, 48h, and 72h of culture. (E) Multiplexed capillary gel electrophoresis coupled to laser-induced fluorescence detection (xCGE-LIF) analysis of 8-aminopyrene-1,3,6-trisulfonic acid (APTS)-labeled N-glycans was performed on AAT recombinantly expressed in CAG-AAT mAM cell line and from human serum to demonstrate physiological glycosilation pattern of transgenic human AAT. Bars display relative signal intensities of N-glycans migrating at the indicated migration time units [MTU]. (F) Elastase inhibition assay of mAM cell supernatants. AAT at different concentrations served as positive control (orange bars). n=3 technical replicates, bars represent mean±SD.

**
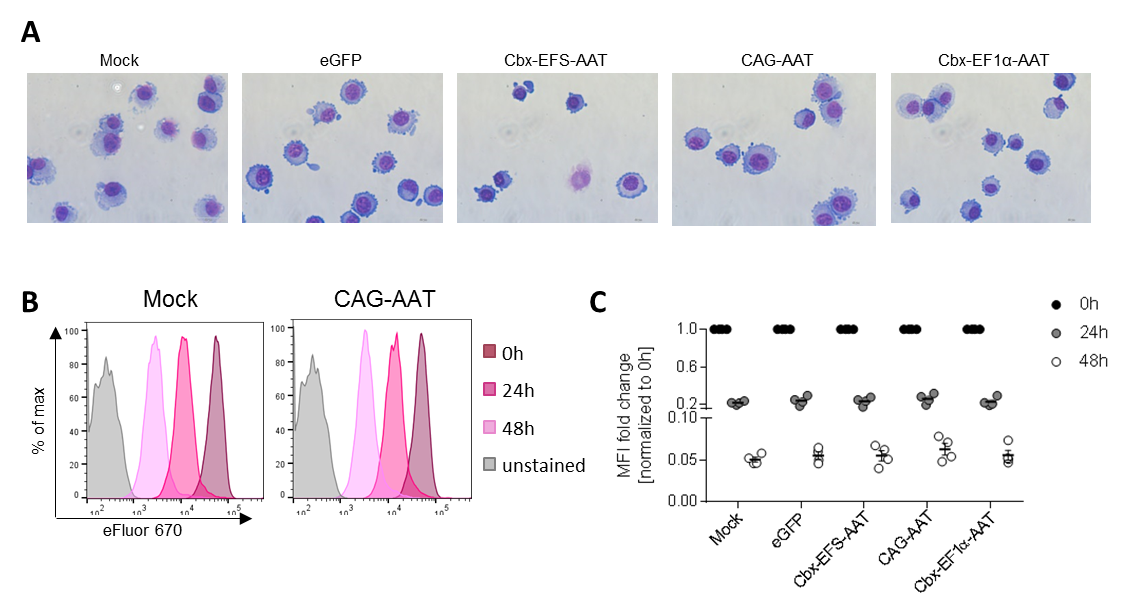
**

**Supplementary Fig. 2. Characterization of transduced mAM cells.** (A) Representative May-Grünwald-Giemsa staining of mAM cell cytospins. Scale bar= 20μm (B) Representative flow cytometric analysis of mAM cells 0h, 24h and 48h after labeling with the eFluor 670 proliferation dye. (C) Fold-change of the median fluorescence intensity (MFI) of the eFluor 670 proliferation dye in mAM cells 0h, 24h and 48h after labeling. MFI values were normalized to the 0h time point. Bars represent mean ± SD, n=4 technical replicates.


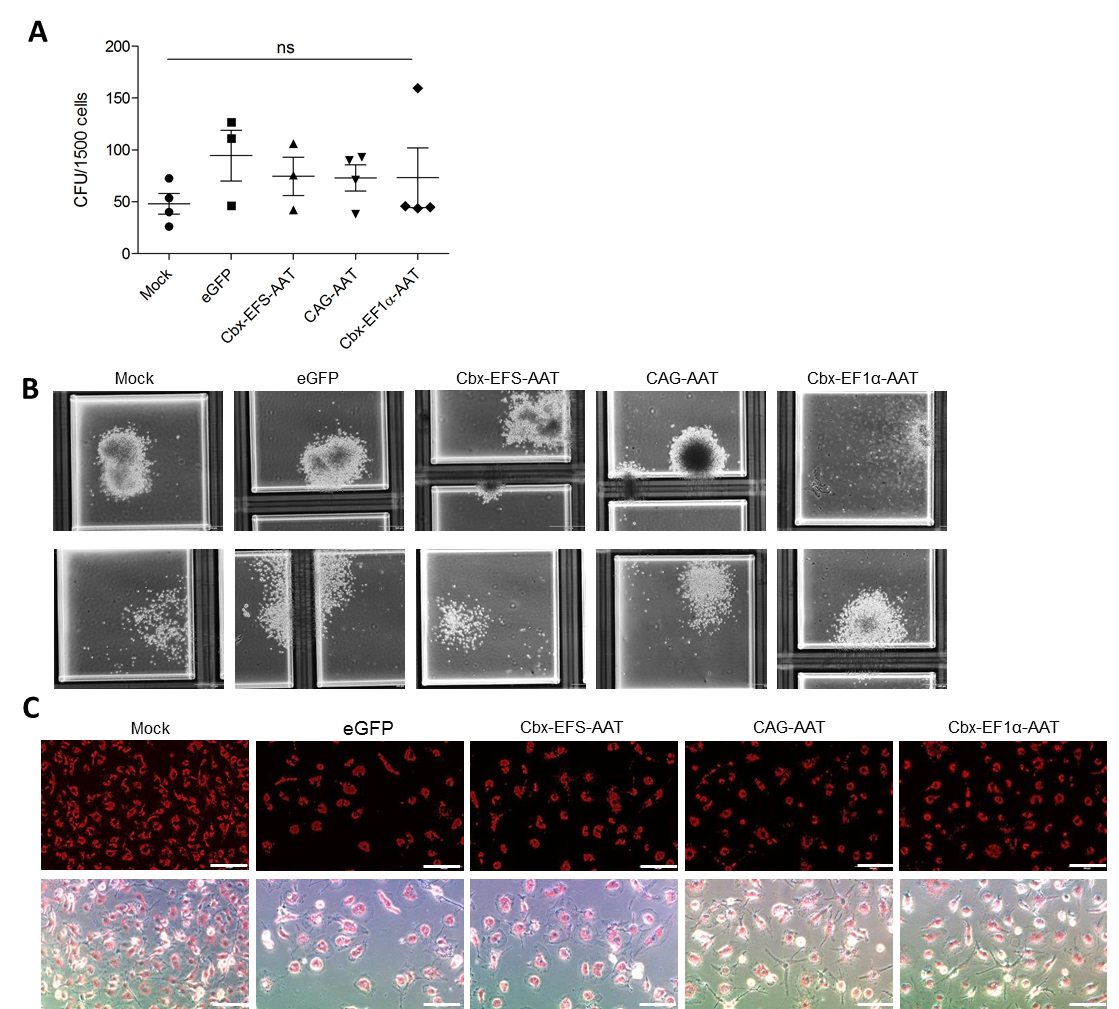


**Supplementary Fig. 3. Functionality of transduced primary murine cells.** (A) Number of colony forming units (CFU) per 1500 input lin^-^ cells in methylcellulose containing EPO (5 IU/ml), IL-3 (10 ng/ml), IL-6 (10 ng/ml), and SCF (50 ng/ml). Statistical analysis was performed using one-way ANOVA with Tukey's post-hoc test. Each point represents an independent experiment. Bars represent mean ± SD. ns = not significant. (B) Representative pictures of colonies in methylcellulose. (C) Representative fluorescent images (upper row) and overlay with phase-contrast pictures (lower row) of murine MΦ incubated with red E. coli pHrodo particles. Scale bar= 100 μm.


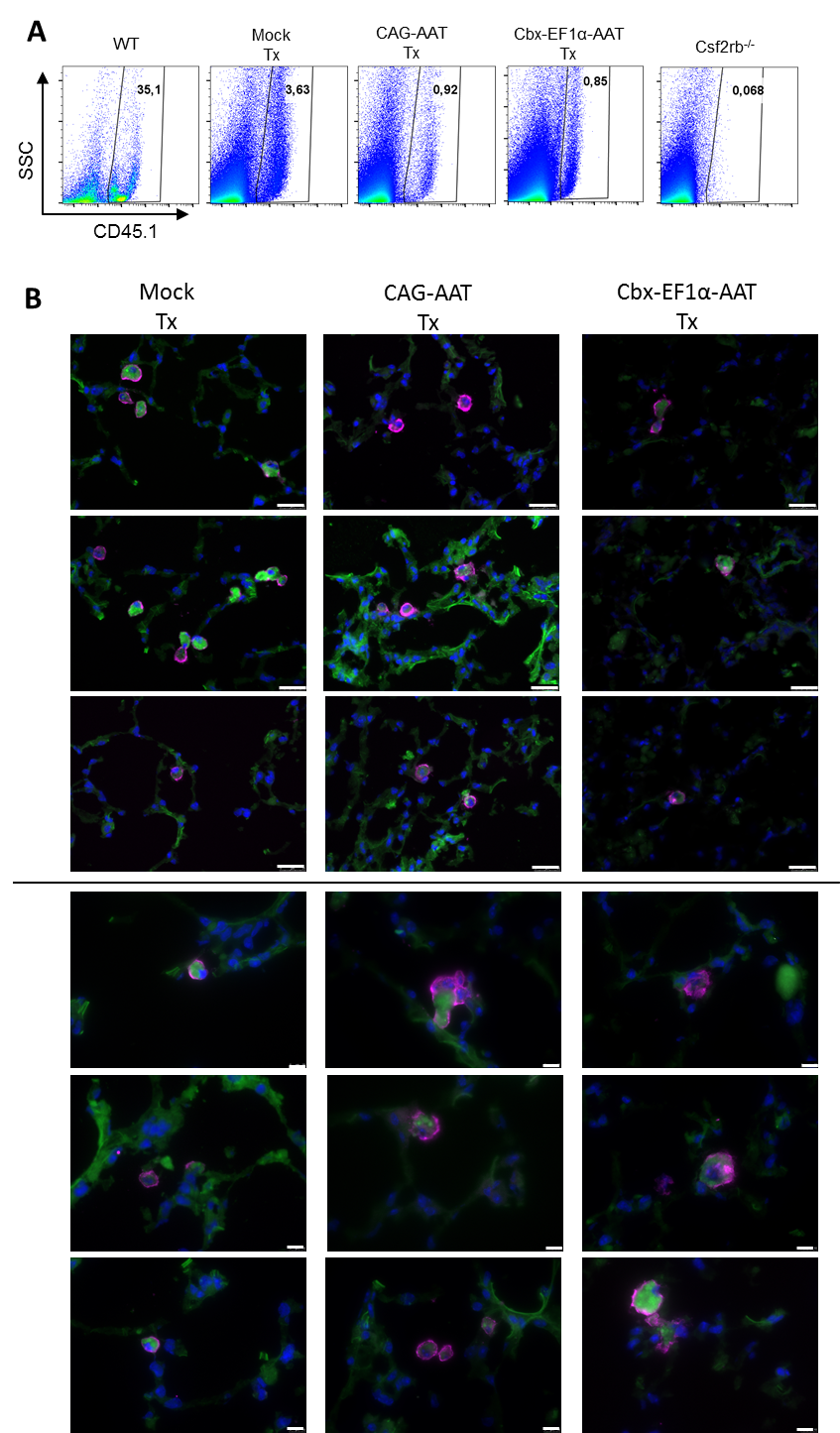


**Supplementary Fig. 4. MΦ engraftment in the lung.** (A) Representative flow cytometric plots demonstrating presence of CD45.1^+^ cells in lung homogenate. SSC = side scatter, WT = wild type, Tx = transplanted. (B) Immunofluorescent staining of lung cryosections depicting the CD45.1 cells (magenta), nuclei (DAPI) and autofluorescent lung structure (green). Scale bar= 25µm (three upper rows), scale bar= 10µm (three lower rows).


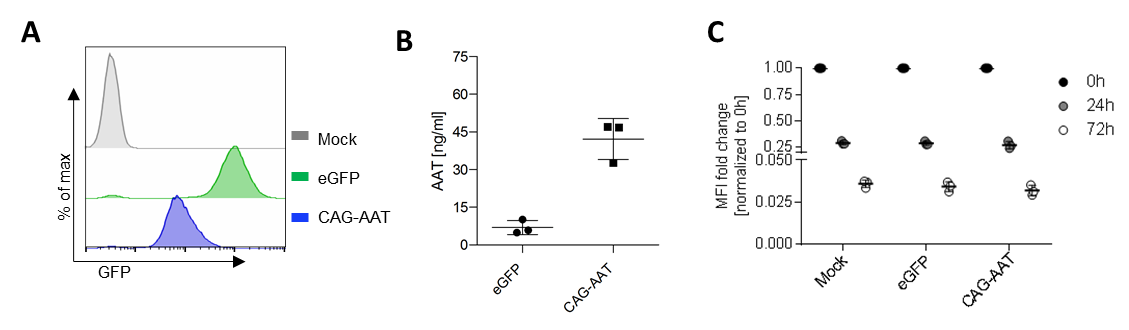


**Supplementary Fig. 5. Characterization of transgenic AAT expression in the U937 human myeloid cell line.** (A) Representative histograms of eGFP expression in mock-, eGFP-, and CAG-AAT transduced U937 cells. The eGFP control vector used here is EFS-IRES-eGFP. (B) ELISA quantification of AAT secretion by transduced U937 cells. (C) Fold-change of the median fluorescence intensity (MFI) of the eFluor 670 proliferation dye in mAM cells 0h, 24h and 48h after labeling. MFI values were normalized to the 0h time point. Bars represent mean ± SD, n=3.

**Supplementary Table 1.** Mass spectometric identification of ~52 kDa protein band isolated from mAM cell culture supernatant.

| **Sample** | **Accession number** | **Protein** | **Peptide matches** |
| --- | --- | --- | --- |
| mAM CAG-AAT | P01009 | Human AAT | 5 |
|  | P01009 | Human AAT | 7 |
|  | P01009-2 | Human AAT | 8 |
